# Harnessed viruses in the age of metagenomics and synthetic biology: an update on infectious clone assembly and biotechnologies of plant viruses

**DOI:** 10.1111/pbi.13084

**Published:** 2019-02-28

**Authors:** Fabio Pasin, Wulf Menzel, José‐Antonio Daròs

**Affiliations:** ^1^ Agricultural Biotechnology Research Center Academia Sinica Taipei Taiwan; ^2^ Leibniz Institute DSMZ‐German Collection of Microorganisms and Cell Cultures Braunschweig Germany; ^3^ Instituto de Biología Molecular y Celular de Plantas (Consejo Superior de Investigaciones Científicas‐Universitat Politècnica de València) Valencia Spain

**Keywords:** plant virus, infectious clone, *Agrobacterium*, transient expression systems, synthetic virus population, crop gene therapy

## Abstract

Recent metagenomic studies have provided an unprecedented wealth of data, which are revolutionizing our understanding of virus diversity. A redrawn landscape highlights viruses as active players in the phytobiome, and surveys have uncovered their positive roles in environmental stress tolerance of plants. Viral infectious clones are key tools for functional characterization of known and newly identified viruses. Knowledge of viruses and their components has been instrumental for the development of modern plant molecular biology and biotechnology. In this review, we provide extensive guidelines built on current synthetic biology advances that streamline infectious clone assembly, thus lessening a major technical constraint of plant virology. The focus is on generation of infectious clones in binary T‐DNA vectors, which are delivered efficiently to plants by *Agrobacterium*. We then summarize recent applications of plant viruses and explore emerging trends in microbiology, bacterial and human virology that, once translated to plant virology, could lead to the development of virus‐based gene therapies for *ad hoc* engineering of plant traits. The systematic characterization of plant virus roles in the phytobiome and next‐generation virus‐based tools will be indispensable landmarks in the synthetic biology roadmap to better crops.

## Introduction

**Figure 1:**
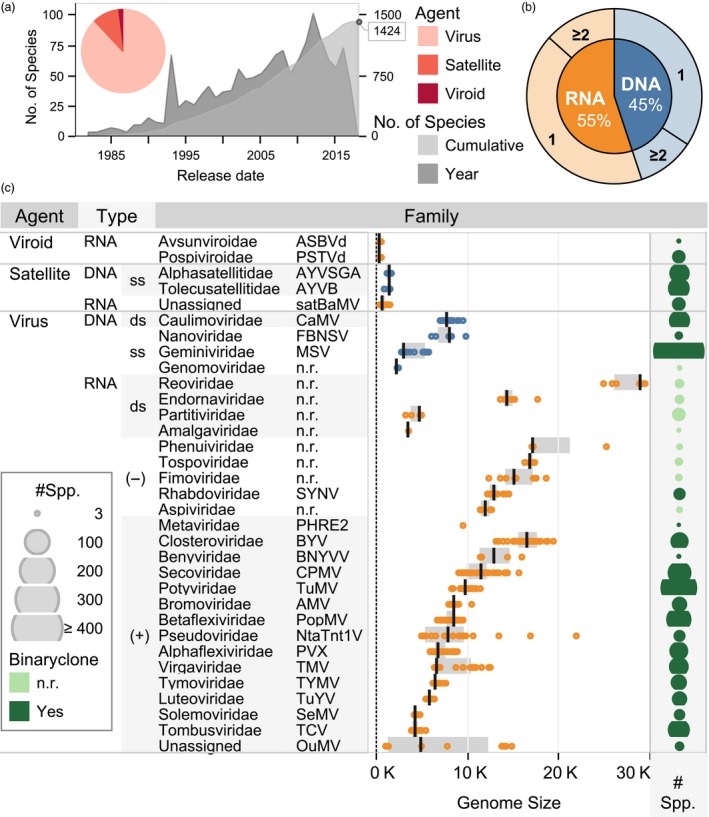
Overview of harnessed plant viruses. (a) Number of species with available genome sequences deposited over the past 35 years (single year and cumulative numbers, left and right axis respectively). Pie chart shows species contribution of viral and subviral agents (viruses, satellites and viroids). (b) Genome composition; DNA or RNA subdivided into mono‐ or multipartite (1 or ≥2 respectively). (c) Viral and subviral agent taxonomy, genome properties and availability of infectious clones in binary vectors (binary clone). Families are subdivided according to types of nucleic acid: ds and ss, double‐ and single‐stranded genomes respectively; (−), negative‐ and negative/positive‐ssRNA and (+), positive‐ssRNA viruses. For each family, sizes of available complete genomes are plotted and median, upper and lower quartiles are shown; on the right, circles show total numbers of International Committee on Taxonomy of Viruses (ICTV) species (#Spp., including those with partial or no genome resources). Acronyms indicate representative species with reported infectious clones in binary vectors: n.r., not reported; ASBVd, *Avocado sunblotch viroid* (Daròs and Flores, 2004); PSTVd, *Potato spindle tuber viroid* (Gardner *et al*., 1986); AYVSGA,* Ageratum yellow vein Singapore alphasatellite* (Idris *et al*., 2011); AYVB,* Ageratum yellow vein betasatellite* (Saunders *et al*., 2000); satBaMV,* Bamboo mosaic virus satellite* RNA (Liou *et al*., 2014); CaMV,* Cauliflower mosaic virus* (Grimsley *et al*., 1986); FBNSV,* Faba bean necrotic stunt virus* (Grigoras *et al*., 2009); MSV,* Maize streak virus* (Grimsley *et al*., 1987); SYNV,* Sonchus yellow net virus* (Wang *et al*., 2015); PHRE2, *Phyllostachys edulis retrotransposon 2* (Zhou *et al*., 2018); BYV,* Beet yellows virus* (Prokhnevsky *et al*., 2002); BNYVV,* Beet necrotic yellow vein virus* (Delbianco *et al*., 2013); CPMV,* Cowpea mosaic virus* (Liu and Lomonossoff, 2002); TuMV,* Turnip mosaic virus* (Lellis *et al*., 2002); AMV,* Alfalfa mosaic virus* (Vlot *et al*., 2001); PopMV,* Poplar mosaic virus* (Naylor *et al*., 2005); NtaTnt1V, *Nicotiana tabacum Tnt1 virus* (Lucas *et al*., 1995); PVX,* Potato virus X* (Baulcombe *et al*., 1995); TMV,* Tobacco mosaic virus* (Turpen *et al*., 1993); TYMV,* Turnip yellow mosaic virus* (Cho and Dreher, 2006); TuYV,* Turnip yellows virus* (Leiser *et al*., 1992); SeMV,* Sesbania mosaic virus* (Govind *et al*., 2012); TCV,* Turnip crinkle virus* (Thomas *et al*., 2003); OuMV,* Ourmia melon virus* (Crivelli *et al*., 2011). Virus taxonomy information (MSL #32; March 12, 2018), sequence accession numbers (VMR 290118) and unassigned satellite species were obtained from the ICTV database (Lefkowitz *et al*., 2018). Genome sequence data and release dates are from NCBI (NCBI Resource Coordinators, 2018) (March 14, 2018). Note that *Metaviridae* and *Pseudoviridae* genome sizes might be incorrect due to inclusion of host sequences in reference accessions.

Viruses are the most abundant biological entities on Earth and can be found in the most diverse environments (Paez‐Espino *et al*., 2016). The advent of high‐throughput sequencing approaches reshaped our perception of plant viral and subviral agents, both in terms of diversity and of integration into phytobiome networks (Maliogka *et al*., 2018; Roossinck *et al*., 2015; Schoelz and Stewart, 2018). The number of genome resources for viral and subviral agents has increased steadily in the past 35 years (Figure 1a). In contrast to prokaryotes and similar to other eukaryotic viromes (Koonin *et al*., 2015), in plants, diversity and abundance of RNA agents exceed those of their DNA counterparts (Figure 1b,c). With these huge sequence datasets in our hands, the quest for ecological and biological characterization of novel viruses appears timely and appropriate (Canuti and van der Hoek, 2014; Massart *et al*., 2017; Roossinck *et al*., 2015). Since the first demonstrations that cloned genome copies of viral and subviral pathogens can initiate plant infections (Ahlquist *et al*., 1984; Cress *et al*., 1983; Howell *et al*., 1980), infectious clones became indispensable tools for characterizing the function of viral components (i.e., transcripts, proteins and non‐translated elements) in the context of virus infections. They also provide a simple, standardized inoculation mode that improves reproducibility and facilitates host–virus interaction studies and crop breeding; this is especially useful for viruses that cannot be transmitted to host plants by mechanical inoculation of virions. Use of infectious clones is part of a proposed framework for the biological characterization of viral agents discovered by high‐throughput sequencing technologies (Massart *et al*., 2017). In one case, full‐length virus clones assembled from 700‐year‐old DNA samples restored the infectivity of an ancient plant virus identified in a metagenomic survey (Ng *et al*., 2014). Establishing reverse genetic systems of newly discovered viruses would help to dissect the contribution of individual viruses identified in mixed infections as well as to provide tools for the study and breeding of neglected and underused crops.

Viruses have been so far an amazing source of genetic elements generally used in all kind of biology approaches (Schoenfeld *et al*., 2010). Infectious clones and virus‐derived devices are applied increasingly for virology‐unrelated fundamental studies as well as for industrial production of biopharmaceuticals and nanomaterials. Plant virus components have been adapted for genetic circuit and biosensor design (Calles and de Lorenzo, 2013; Cordero *et al*., 2018; Fernandez‐Rodriguez and Voigt, 2016; Gao *et al*., 2018; Gray *et al*., 2010; Stein *et al*., 2017; Wehr *et al*., 2006), and unprecedented parts for plant synthetic biology may be about to be discovered from currently uncharacterized viruses.

In this review, we present advances in molecular and synthetic biology that hasten infectious clone assembly, thus lessening a major technical restraint of plant virology. *Agrobacterium* strains are used to transform plant cells by transferring into hosts the T‐DNA cassette of binary vectors, and can be further exploited to launch virus infections in a method known as agro‐inoculation or agro‐infection (Grimsley *et al*., 1986). Due to their flexibility and efficiency, we focus on the construction and use of binary infectious clones, that is, T‐DNA plasmids suitable for propagation in *Escherichia coli* and *Agrobacterium*, and including virus genome copies that can be delivered to plants by agro‐infection. We further discuss current biotechnology applications of plant viruses and virus‐derived constructs in general, and explore recent developments in the phage and medical research fields that could inspire future plant virus‐based strategies for improvement of crop traits.

## *Agrobacterium*‐mediated inoculation of plant viruses

Plants can be inoculated using DNA or RNA infectious molecules, which can be obtained from infected plant samples or plasmid‐based infectious clones. These molecules represent virus evolutionary snapshots that can be stably propagated in bacteria to produce large amounts of inoculum and provide a basis for reverse genetic studies of plant viruses. For RNA viruses, cDNA copies of virus genomes can be driven by bacteriophage promoters and transcribed *in vitro* to generate infectious RNA genomes. Plasmid clones of DNA viruses and RNA viruses whose cDNA genomes are driven by promoter sequences active in plants can be inoculated directly to plants by physical methods, that is, with the help of abrasives or biolistic devices (Nagyová and Subr, 2007).

*Agrobacterium* can be used for stable or transient transformation of plant cells with exogenous DNA molecules (Krenek *et al*., 2015). A major discovery in plant virology was the demonstration that *Agrobacterium* can launch virus infections by treatment of host leaves with bacterial strains that harbour infectious clones of plant viruses (Grimsley *et al*., 1986). Briefly, single or multiple copies of virus genomes are inserted between the T‐DNA left and right borders of a plasmid suitable for *Agrobacterium* replication. Once bacteria contact plant tissues, T‐DNA cassettes are transferred into host cells, and host transcription and translation of T‐DNA sequences trigger synthesis of the viral components needed to start autonomous infections. T‐DNA cassettes do not require stable integration in host genomes, and transient expression is sufficient to achieve plant infection. This feature, together with *Agrobacterium* promiscuity and its extensive host range (Lacroix *et al*., 2006), made agro‐inoculation a method successfully applied to dicot and monocot plants (Bhat *et al*., 2016; Grimsley *et al*., 1986, 1987; Liou *et al*., 2014; Lu *et al*., 2012; Scofield and Nelson, 2009) as well as herbaceous and woody hosts, including citrus, grapevine or apple (Cui *et al*., 2018; Dawson and Folimonova, 2013; Kurth *et al*., 2012; Velázquez *et al*., 2016; Zhang and Jelkmann, 2017).

Given its simplicity and convenience, agro‐inoculation is thus the most efficient and universal way of delivering to plants DNA or RNA viruses and subviral agents, such as viroids and satellites (Peyret and Lomonossoff, 2015). Binary infectious clones have been reported for many virus, satellite and viroid species with mono‐ or multipartite genomes, and which belong to phylogenetically diverse families (Figure 1c).

Assembly of binary infectious clones is relatively straightforward for DNA viruses, since their genomes can be subcloned directly into plasmid vectors and they harbour elements needed to drive expression of viral genes in plant. In contrast, cloning RNA viruses requires additional manipulation steps, for example, cDNA synthesis, and inclusion of promoter, terminator sequences that regulate expression of viral components in plants (Nagyová and Subr, 2007).

## Advanced methods for binary infectious clone assembly

**Figure 2:**
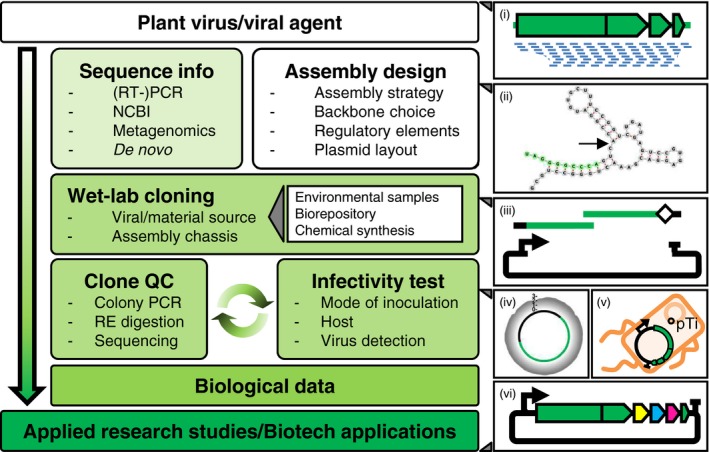
Workflow for infectious clone assembly of plant viruses. Once viral and subviral agents are identified, sequence analysis, assembly design and wet‐lab cloning are carried out to obtain virus clones, that is, plasmid vectors with full‐length copies of virus genomes. After clone quality controls (QC) and infectivity tests, infectious clones are used for biological characterization of novel viruses. Infectious clones might have uses in applied research studies, for crop breeding, and development of biotechnological applications. Right, and from top to bottom, representative workflow steps are depicted: (i) virus identification by short‐read sequencing; (ii) design of a regulatory element (i.e. ribozyme) to improve inoculation efficiency; (iii) one‐step, overlap‐based assembly of virus genome and regulatory elements into a binary vector; (iv) validation of full‐length clones by Illumina sequencing; and (v) infectivity tests by *Agrobacterium*‐mediated inoculation (pTi, disarmed tumour‐inducing plasmid); (vi) viral vector engineering for heterologous protein expression in plants. Adapted from Pasin *et al*. (2018).

The main goal of infectious clone assembly is the construction of plasmid vectors that harbour a faithful copy of virus genomes, which in appropriate conditions can launch a plant infection that mimic natural ones. Detailed step‐by‐step protocols have been described elsewhere (Nagata and Inoue‐Nagata, 2015; Peremyslov and Dolja, 2007), and a workflow summary is shown in Figure 2. Here, we review recent improvements that have allowed one‐step, streamlined assembly of binary infectious clones with no intermediate subcloning steps.

For decades, the most common approaches to assembly of DNA constructs and infectious clones have taken advantage of restriction endonuclease specificities to create compatible ends that were joined using DNA ligases. The presence or lack of restriction sites in vector backbone and viral sequences were major constraints to the assembly of large and multiple inserts (Nakahara *et al*., 2015). Cloning methods have been developed to overcome these limitations, thus allowing high‐throughput assembly of DNA constructs (reviewed in (Chao *et al*., 2015; Liu *et al*., 2013)). Recombinase‐based technologies, such as Gateway, Creator and Echo cloning and SIRA, are widely adopted for building of gene constructs due to their high efficiency, flexibility and comprehensive plasmid collections (Karimi *et al*., 2007). Current recombinase‐based technologies are not scar‐free, as recombination sequences are retained in final assemblies. Cloning scars might alter virus viability and infectivity, especially when multiple fragments are joined to span the entire virus genome sequence. Not surprisingly, in recent years, plant virologists have increasingly adopted seamless cloning strategies (Table 1). These include type IIS restriction endonuclease‐ and overlap‐based methods, which have the ability to join 2–10 fragments in a predetermined order to yield final constructs lacking assembly scars, that is, seamless. Type IIS restriction enzymes cut DNA outside their recognition site and produce an overhang of 1–5 nucleotides, which can be chosen to generate a ligation product free of unwanted sequences. In Golden Gate cloning, a one‐pot mix of type IIS endonucleases (i.e. Bsal or BsmBl) and a DNA ligase, allows simultaneous digestion and assembly of multiple fragments (Engler *et al*., 2008). Despite its advantages, use of Golden Gate has not been yet reported for construction of infectious clones. As traditional restriction enzyme‐based methods, fragments with internal instances of the type IIS endonuclease recognition site are not suitable for Golden Gate, which complicates assembly of large virus genomes. Compared to BsaI or BsmBI, use of 7‐nt type IIS cutters such as SapI and AarI cope better with large assemblies (Andreou and Nakayama, 2018), and could be a better choice for standardized high‐throughput assemblies of infection clones.

**Table 1:** Representative seamless cloning methods suitable for assembly of binary infectious clones

Cloning system	Components	Use†
Golden Gate	Type IIS endonuclease, DNA ligase	n.r.
GeneArt seamless cloning kit	Proprietary	Wieczorek *et al*. (2015)
In‐Fusion	Vaccinia virus DNA polymerase	Wang *et al*. (2015)
Gibson assembly	T5 DNA exonucluease, Phusion DNA polymerase, Taq DNA ligase	Blawid and Nagata (2015)
NEBuilder HiFi DNA assembly	Proprietary	Pasin *et al*. (2017)
*In vivo* yeast assembly	Plasmid vectors with yeast origin	Youssef *et al*. (2011)
*In vivo* bacterial assembly (recombineering)	*E. coli* RecET‐/Redαβγ‐expressing strains	n.r.

† Use for infectious clone assembly in binary vectors: ToTV, *Tomato torrado virus* (Wieczorek *et al*., 2015); SYNV, *Sonchus yellow net virus* (Wang *et al*., 2015); TBMT, *Tomato blistering mosaic virus* (Blawid and Nagata, 2015); UCBSV, *Ugandan cassava brown streak virus* (Pasin *et al*., 2017); ACLSV, *Apple chlorotic leaf spot virus* (Youssef *et al*., 2011); n.r., not reported.

Overlap‐based methods are very versatile, extremely flexible and, by solving major constraints of restriction enzyme use, they are revolutionizing the construction of virus infectious clones. The GeneArt seamless cloning kit (Thermo Fisher Scientific, Waltham, MA) contains a proprietary enzyme mix with exonuclease activity that allows one‐step assembly of fragments with 15‐bp homology; it has been used to build agroinfectious clones of genera *Torradovirus* (Wieczorek *et al*., 2015) and *Cucumovirus* (Wrzesińska *et al*., 2016). The In‐Fusion system (Takara Bio, Mountain View, CA) relies on the vaccinia virus DNA polymerase, which attacks linear DNA to expose 5′ ends and promote annealing of fragments with 15‐bp sequence overlap (Irwin *et al*., 2012). Molecules assembled by In‐Fusion are not covalently joined and are sealed *in vivo*. A potyvirus infectious clone for *in vitro* transcription (Tuo *et al*., 2015) and a binary vector for agro‐inoculation of a trichovirus have been generated by this method (Zhang and Jelkmann, 2017). In‐Fusion was also used to engineer the first binary vector for plant delivery of a negative‐stranded RNA virus (Wang *et al*., 2015). Notwithstanding, the one‐pot, one‐step isothermal assembly method described by Gibson *et al*. (2009) is by far the most popular. During Gibson assembly (SGI‐DNA, La Jolla, CA), an exonuclease exposes 3′ ends of linear DNA; then, fragments that share terminal overlapping homologies anneal and prime a DNA polymerase which fills overhang gaps; finally, a DNA ligase seals the nicks. In contrast to In‐Fusion, Gibson assembly yields closed circular DNA molecules. Applied for reverse genetic studies of potyvirus clones (Bordat *et al*., 2015; Pasin *et al*., 2014), Gibson assembly has been used to generate binary infectious clones of members of genera *Tymovirus* (Blawid and Nagata, 2015), *Carlavirus* (Carvalho *et al*., 2017), *Comovirus* (Bijora *et al*., 2017), *Potyvirus* (Rose and Maiss, 2018), *Polerovirus* (Wetzel *et al*., 2018), *Benyvirus* (Laufer *et al*., 2018), *Tobamovirus* (Vasques *et al*., 2018) and *Begomovirus* (Ferro *et al*., 2019). Gibson assembly versions with improved fidelity are commercially available (e.g. the NEBuilder HiFi DNA assembly mix; New England Biolabs, Ipswich, MA), and have been used to generate binary infectious clones of ssRNA as well as ds‐ and ssDNA viruses of genera *Potyvirus*,* Ipomovirus*,* Tobamovirus*,* Caulimovirus* and *Turncurtovirus* (Pasin *et al*., 2017, 2018).

As an alternative to *in vitro* assembly methods, circular plasmids can be produced *in vivo* by the cell endogenous homologous recombination machinery. Homologous recombination occurs naturally in yeast with high efficiency and fidelity, and has been used for decades to construct plasmids from DNA fragments containing homologous regions. More recently, co‐transformation of yeast cells with 25 different overlapping fragments allowed correct assembly of a 590‐kb molecule (Gibson *et al*., 2008). This finding highlights the extreme potential of *in vivo* yeast assembly, which has been used for target mutagenesis of a polerovirus clone (Liang *et al*., 2004), and later to assemble binary infectious clones of members of genera *Trichovirus*,* Potyvirus* and *Mandarivirus* (Cui *et al*., 2018; Sun *et al*., 2017; Youssef *et al*., 2011). Bacteria provide high transformation efficiency, plasmid yields and rapid growth rates, but homologous recombination efficiencies reported in *E. coli* are orders of magnitude lower than those of yeast. Unsatisfactory results of bacterial homologous recombination prompted the design of new *in vivo* assembly strategies. Expression of RecET proteins from the Rac prophage or the Redαβγ from lambda phage greatly improves homologous recombination in *E. coli* (Murphy, 1998; Zhang *et al*., 1998). These *in vivo* recombination systems, known as recombineering, are frequently used to engineer bacteriophage genomes (Lemire *et al*., 2018). Highly efficient homologous recombination between linear DNA molecules was obtained by direct transformation of *E. coli* strains expressing the RecET proteins (Fu *et al*., 2012). Although it has yet to be reported, use of recombineering and enhanced RecET‐expressing bacteria are promising approaches for assembly of plant virus infectious clones in binary vectors.

## Improving clone stability…

Plant viruses are often recalcitrant to molecular cloning. Sequence alterations, such as point mutations and deletions, might arise during clone assembly and propagation in bacteria (Bedoya and Daròs, 2010; Satyanarayana *et al*., 2003). Spontaneous acquisitions of DNA inserts and transposons that disrupt viral genes have also been reported (Donson *et al*., 1993; González *et al*., 2002; Tran *et al*., 2019; Yount *et al*., 2000). These have been linked to toxicity of unwanted viral expression products in bacterial hosts, and several approaches were therefore designed to tackle instability issues. Although inconvenient for routine cloning procedures, assembly chassis that better tolerate toxic, difficult constructs, such as yeast and *Agrobacterium*, have been used for binary clone assembly (Sun *et al*., 2017; Tuo *et al*., 2017; Youssef *et al*., 2011). Better choices for repeated plasmid manipulations are *E. coli* strains suitable for cloning unstable DNA, that is, Stbl2 and Stbl4 (ThermoFisher), and SURE2 (Agilent Technologies, Santa Clara, CA). Reduction in *E. coli* incubation temperatures (e.g. from 37 °C to 25–30 °C) slows bacterial growth rates and improves maintenance and propagation of problematic constructs. These approaches nonetheless palliate rather than solve plasmid instability. A popular strategy used to obtain stable infectious clones involves interruption of viral genes with eukaryotic introns that contain multiple stop codons; these avoid accumulation of undesired viral proteins and the consequent toxicity in bacteria (Johansen and Lund, 2008). After plant inoculation, introns are removed by splicing, viral sequences are reconstituted *in vivo* and infections initiate. Introns were successfully used to stabilize infectious clones of diverse viruses including large positive‐strand RNA viruses from the *Potyviridae*,* Closteroviridae* and *Coronaviridae* families (Ambrós *et al*., 2011; González *et al*., 2002; Johansen, 1996; López‐Moya and García, 2000). Insertion of multiple introns may be required to disrupt putatively toxic genes and stabilize clones (Bukovinszki *et al*., 2007; Gao *et al*., 2012). For the same purpose, targeted introduction of mutations that change translation frame or remove cryptic bacterial promoters and ribosomal binding sites from viral sequences has also been applied to improve clone stability (Chikh Ali *et al*., 2011; Pu *et al*., 2011; Satyanarayana *et al*., 2003). Although powerful, these strategies might require intermediate subcloning steps that complicate assembly designs.

In many cases, rational choice of vector backbones, regulatory elements and component layout is a sufficient and key factor in the assembly of stable infectious clones with intron‐free, unmodified copies of full‐length virus genome sequences (Bedoya and Daròs, 2010). Use of binary vector backbones with reduced copy number origins lessens foreign DNA loads and potential bacterial toxicity of the viral sequences. Binary vectors with single‐, low‐ or medium‐copy origins are available (Hamilton *et al*., 1996; Pasin *et al*., 2017; Xiang *et al*., 1999) and have been used to generate infectious clones of members of families with large genomes, such as *Potyviridae*,* Rhabdoviridae* and *Closteroviridae* (Ambrós *et al*., 2011; Lellis *et al*., 2002; Pasin *et al*., 2017, 2018; Prokhnevsky *et al*., 2002; Shi *et al*., 2016; Wang *et al*., 2015). Choice of binary vector origin was also shown to affect *Agrobacterium* transformation efficiency in both stable transformation and transient expression assays (Pasin *et al*., 2017; Zhi *et al*., 2015).

Cloning cassettes often include reporter genes under strong bacterial promoters (e.g. *lacZ*α for white/blue screens). Improper removal of reporter genes and their regulatory elements could lead to the inadvisable result that the entire virus genome is constitutively expressed in the bacterial host. Insertion of virus genome cassettes in reverse orientation to genes needed for plasmid maintenance (i.e. resistance markers, replication proteins) helps prevent viral gene transcription, translation and consequent toxicity in bacteria (Bedoya and Daròs, 2010). Flanking inserts with bacterial terminators avoids transcriptional read‐through from adjacent sequences and increases plasmid stability. Binary vectors with strong synthetic bacterial terminators up‐ and downstream of the T‐DNA cassette have been described, and used for assembly of clones with intron‐free copies of virus genomes (Pasin *et al*., 2017, 2018).

A stable full‐length cDNA clone of Zika virus was recently assembled into the linear vector pJAZZ (Annamalai *et al*., 2017; Godiska *et al*., 2010). Although not reported to date, linear plasmids may also be used to generate stable infectious clones of plant viruses.

Finally, basic microbiology skills are helpful for obtaining full‐length infectious clones. A mixture of small and large colonies can appear during agar plate selection of transformed bacteria. As large colonies often contain partial or rearranged plasmids, small colonies should be selected for subsequent analysis (González *et al*., 2002). Bacteria with correct plasmids can have slow growth rates due to the burden associated with propagation of large inserts and/or with leaky expression and toxicity of viral genes (Satyanarayana *et al*., 2003). Long incubation times allow slow‐growing colonies to appear and increase the chance of obtaining positive clones. For the assembly of novel infectious clones and as a rule of thumb, we thus recommend incubating *E. coli* plates at 30 °C (see above) for 36–72 h and that picking large colonies should be avoided. Long incubation times might also be required during *E. coli* growth in liquid cultures and infectious clone propagation in *Agrobacterium*.

## …and inoculation efficiency

During cloning design, additional factors should be considered for success in obtaining viable infectious clones and to improve their inoculation efficiency. Knowledge of complete virus genome or reliable consensus sequences is highly desirable; for linear genomes exact, terminal sequences should be determined, since authentic termini are usually critical for virus viability. This holds true for each subgenomic component of multipartite viruses (Figure 1b), as partial virus reconstitution can preclude or alter infectivity and biological properties (Grigoras *et al*., 2009). Virus propagation in experimental plant species can drive a rapid adaptive evolution altering virus host range (Kurth *et al*., 2012); virus‐infected natural hosts should be preferred as a starting material for infectious clone assembly. Retrieval of virus samples from public biological resource centres can help when original materials are not available (Box 1).

Box 1 **Biological resource centres for plant virologists**

Virus isolates are important references in taxonomic research and constitute the basis of fundamental as well as applied research studies. These include the construction of full‐length infectious clones to analyse virus component functions, virus–host and virus–vector interactions. Infectious clones might also replace natural isolates in breeding screens to identify plant genotypes with enhanced virus resistance. Characterized virus isolates are indispensable for the development and validation of detection methods of any kind (biological, serological, molecular) and subsequently form the basis for the production of required positive controls for routine testing. Moreover, multiple isolates with diverse geographic origin and hosts allow the study of species diversity.

The vast majority of virus isolates are maintained by individual scientists at research institutes or in local working collections of diagnostic laboratories. Non‐public collections often include a small number of species, but they may have considerable depth and specialization. Unfortunately, such collections might not be “visible”, might be difficult to access and/or have limited financial and human resources. There are many public microorganism collections. In contrast to bacteria or fungi, it is not mandatory to deposit type isolates of newly described virus species. To ensure their long‐term availability, it is the responsibility of each scientist to deposit isolates in curated virus collections with the infrastructure and sustainable funding to preserve them. Only a few biological resource centres maintain virus collections with worldwide, publicly accessible information about their resources and availability (Box 1 Table). In recent decades, the implementation of quality management systems and accreditation of biological resource centres according to international standards such as ISO 17025 or ISO 17034 have gained importance and guarantee a comprehensive, unambiguous quality standard for their respective activities and reference materials.

**Table** A selection of biological resource centres providing plant virus materials

**Table:** 

Center	Country	Link	Available resources
Agriculture Agri‐Food Canada	CA	Public portal under construction	Virus isolates primarily stored as freeze‐dried tissue and some in live plants. Requests to Michael Bernardy (mike.bernardy@canada.ca)
ATCC	USA	http://www.atcc.org/	Virus isolates stored as freeze‐dried tissue, some as plasmids (partial genome clones) and antisera
Leibniz Institute DSMZ	DE	http://www.dsmz.de/	Virus isolates primarily stored as freeze‐dried tissue and some in live plants, serological positive controls, nucleic acid extracts and antisera
NARO Genebank	JPN	http://www.gene.affrc.go.jp/	Virus isolates for research and educational purposes, results must be reported to NARO Genebank
Plant Virus GenBank	KOR	http://knrrb.knrrc.or.kr/index.jsp?rrb=pvgb	Virus isolates, plasmid clones and antisera. Limited English information
Q‐bank	Several	http://www.q-bank.eu/Virus/	Q‐bank is only a database, but provides information on virus isolates and contact details, where, and in what form they can be obtained
World Federation for Culture Collections	Several	http://www.wfcc.info/ccinfo/	A worldwide directory of all registered culture collections. Not limited to plant viruses

Plasmids with tandem genome repeats are needed to rescue viruses and subviral agents with circular components (Grimsley *et al*., 1986; Kushawaha and Dasgupta, 2018; Sanjuán and Daròs, 2007). Once delivered to plants, some viruses tolerate ancillary sequences derived from binary vector backbones, and genomes with authentic termini can be recovered after initial rounds of virus replication. Promoters designed to initiate transcription at the authentic 5′ end of virus cDNA and inclusion of synthetic ribozymes help remove non‐viral nucleotides and improve infections of some RNA viruses (Turpen *et al*., 1993; Wang *et al*., 2015). The hepatitis delta virus antigenome ribozyme self‐cleaves at its 5′ terminus and can release exact 3′ end of viral RNAs (Mörl *et al*., 2005; Wrzesińska *et al*., 2016). Proper design of hammerhead ribozymes allows the production of RNA genomes with the desired 3′ as well as 5′ ends. Hammerhead ribozymes show robust activity in diverse *in vivo* systems (Lou *et al*., 2012), are small and easily incorporated in amplification primers (Jarugula *et al*., 2018; Pasin *et al*., 2018; Peremyslov and Dolja, 2007). Viral sequences might interfere with ribozyme folding, leading to reduced or no cleavage. RNAfold is a useful predictor (Lorenz *et al*., 2011), and misfolding of hammerhead ribozymes can be solved by extending their 3′ end with nucleotides complementary to the virus genome terminus. A guide to ribozyme design can be found elsewhere (Mörl *et al*., 2005).

Apart from stabilizing the plasmid, single or multiple introns can act as expression enhancers and have been shown to improve the initiation of viral replication in plants. It can be beneficial to remove features that might induce abnormal processing events, such as cryptic splice sites, nucleotide secondary structures or polyadenylation signals; this should be determined empirically, however, and regarded as a viral vector optimization phase (Marillonnet *et al*., 2005).

Copies of viral sequences are usually obtained by *in vitro* amplification reactions. Short thermocycles and use of high‐fidelity enzymes reduce accumulation of incidental mutations; error rates of commercial DNA polymerases have been recently assessed (Potapov and Ong, 2017). Once binary vectors with virus genome copies are obtained, their cloned sequences should be determined. The dideoxy chain‐termination method (Sanger) currently provides reads slightly <1 kb in length. In most cases, covering entire virus genome inserts can be tedious and time‐consuming, since Sanger limited throughput forces to adopt primer walking strategies, involving several sequencing primers and reactions. A sequencing‐by‐synthesis approach (Illumina) and an automated read assembly pipeline were recently applied to validate binary infectious clones of members of the *Virgaviridae*,* Geminiviridae*,* Caulimoviridae* and *Potyviridae* families (Pasin *et al*., 2018). Despite Illumina short‐sequence reads, it was possible to correctly assemble the *de novo* complete virus genomes and vector backbones with no requirements for reference sequences, custom‐made primer design or data analysis.

*Agrobacterium* is used as a delivery chassis to assess infectivity of virus clones in binary vectors. The *recA*‐deficient strains such as UIA143 and AGL1 are unable to carry out homologous recombination functions and are expected to stabilize large constructs (Farrand *et al*., 1989; Lazo *et al*., 1991). It is currently not known whether these strains confer any agro‐infection improvement over their parental strains. Hypervirulent strains of *Agrobacterium,* such as the succinamopine‐type EHA105 and AGL1, improve transformation efficiencies (Hellens *et al*., 2000; Zhi *et al*., 2015), and might increase agro‐inoculation success rates. The chrysopine‐type strain Chry5 was originally isolated from chrysanthemum (Shao *et al*., 2018) and its derivative strain CryX was reported to have agro‐inoculation efficiencies 100–1000 times higher than those of commonly used *Agrobacterium* strains (Roemer *et al*., 2015). A set of plant species can be tested as experimental hosts, especially if the virus natural host is unknown or difficult to obtain and raise. In the model plant *Nicotiana benthamiana*,* Agrobacterium* cultures are easily delivered by syringe infiltration to the leaf surface. Leaves of crop and non‐model plants might not be suitable for conventional infiltration; agroinfection can be greatly improved by mechanical wounding and use of abrasives, detergents or surfactants (Azhakanandam *et al*., 2007; Giritch *et al*., 2013; Gleba *et al*., 2014; Grimsley *et al*., 1986; Hahn *et al*., 2015; Krenek *et al*., 2015). Viruses might be asymptomatic, vertically inherited in host plants and lack cell‐to‐cell movement (Roossinck, 2010); these aspects should be taken into account during experimental design and result evaluation.

## From clone assembly to *de novo* synthesis

The potential of synthetic biology is reflected by demonstrations that artificial and functional genomes can be generated by *de novo* synthesis and assembly. Due to their limited genome sizes, first proof‐of‐principle results were derived from synthesis of artificial replicons of viral pathogens (Schindler *et al*., 2018). Advances in phage engineering and assembly reports of synthetic eukaryotic viruses up to a 212‐kb genome (Noyce *et al*., 2018; Schindler *et al*., 2018), whose size substantially exceeds that of the largest plant virus (Figure 1c), leads us to wonder whether future generations of virologists will need any molecular cloning skill at all. Current chemical DNA synthesis prices are not compatible with routine cloning, although a sharp price drop catalysed by new technology developments is expected in the near future (Schindler *et al*., 2018). In the plant virology field, infectious clones have been reported for delivery of tobamovirus, tombusvirus and potexvirus genomes synthesized entirely from scratch (Bouton *et al*., 2018; Cooper, 2014; Lovato *et al*., 2014). Due to errors in the reference sequence used, a first version of the synthetic tobamovirus genome was not infectious. Sequence changes were needed to restore clone infectivity (Cooper, 2014), further emphasizing the importance of faithful reference or consensus sequences in the assembly of infectious clones. Technological progress is likely to overcome this limitation. A pipeline described to filter nucleotide variants and determine a putative consensus was used to synthesize a 29.7‐kb infectious cDNA clone of a coronavirus (Becker *et al*., 2008). Populations of viruses can exist as a quasispecies, that is, a haplotype collection, whose consensus sequence might not be infectious. Third‐generation sequencers produce (extra) long reads that allow reconstruction of full‐length viral haplotypes (Ameur *et al*., 2019; Pagán and García‐Arenal, 2018). Chemical synthesis of selected haplotypes would generate variant clones with faithful linkage between mutations and that are more likely to be infectious than consensus clones.

Cell‐free cloning approaches have been developed to obtain infectious constructs of plant DNA and RNA viruses (Fakhfakh *et al*., 1996; Haible *et al*., 2006; Jailani *et al*., 2017; Youssef *et al*., 2011). Uncloned genome copies are obtained by *in vitro* amplification and reaction products are used directly to inoculate plants by rubbing or biolistic delivery. Generation of infectious genome copies by *in vitro* amplification provides a quick means for preliminary studies, but it is likely not suitable for extensive reverse genomic studies. Drastic decreases in DNA synthesis costs will allow the manufacture of entire virus genome libraries and could fuel a revival of uncloned constructs for plant inoculation.

## Virus‐induced plasticity and physiology of plant infections

Plants, their associated micro‐ and macroscopic organisms together with the environmental space they occupy constitute the phytobiome. By facilitating detection and identification of nucleotide sequences, high‐throughput sequencing technologies enable unprecedented opportunities for plant viral and subviral agent discovery (Maliogka *et al*., 2018; Roossinck *et al*., 2015; Schoelz and Stewart, 2018; Wu *et al*., 2012). Compared to virus discovery, however, technological advances for efficient biological characterization of newly identified and known viruses lag behind, thus hampering systematic evaluation of virus‐derived effects on their hosts and other taxa within the phytobiome (Massart *et al*., 2017; Schoelz and Stewart, 2018). Communication networks of phytobiome taxa can result in unforeseeable and surprising outputs that, once properly understood, might be hijacked to improve crop physiological and agronomic traits (Schoelz and Stewart, 2018). The roles of fungi and bacteria in promoting plant fitness and their impacts on plant phenotypic plasticity are well documented (Goh *et al*., 2013). Despite their minimal genomes, viruses can have dramatic effects on plant physiology and plasticity; thus, virus infections may be seen as a means to unleash the phenotypic potential of a defined plant genotype. Plant viruses are known to manipulate their hosts and insect vectors to promote viral transmission (Groen *et al*., 2017); however, virus‐induced plasticity and its beneficial effects on plant traits are insufficiently studied. Desirable virus‐induced phenotypes include drought or cold tolerance, increased resistance to some pathogens and the renowned flower colour breaking that spurred breeding of tulip genotypes that mimic virus infection phenotypes even in the absence of pathogens (Perrone *et al*., 2017; Ramegowda and Senthil‐Kumar, 2015; Schoelz and Stewart, 2018).

Due to its efficiency and universality, agro‐infection can be seen as a standardized method to deliver and dissect physiological and phenotypic outputs of a plant genotype upon virus infection. Similar to other research fields (Großkinsky *et al*., 2018; Houle *et al*., 2010), future use of phenomics for comprehensive characterization of virus‐induced physiology and phenotypic variations, in standard and extreme environmental conditions, will facilitate recognition of virus contributions to the phytobiome.

## Cutting‐edge applications of plant viruses

Although fortuitous, the use of viruses to enhance the beauty of ornamental plants can be considered the first recorded application of plant viruses (Valverde *et al*., 2012) (Table 2). Intentional agricultural uses of plant viruses were also reported. Natural virus strains and engineered mutants with a mild or attenuated symptomatology can safeguard plants from more severe infections through a phenomenon called cross‐protection. Described almost 100 years ago, cross‐protection has been applied to promote the health of crops, including cucurbits, papaya and citrus (Ziebell and Carr, 2010). Commercial use of viroids is approved in United States to induce desired dwarfing in citrus trees and increase yields per land surface unit (Vidalakis *et al*., 2011). Plant viruses have been used as herbicides to control invasive weeds by inducing lethal hypersensitive reactions (Harding and Raizada, 2015). The very first approval for open field use of a plant virus as a bioherbicide was granted in 2015. Tobacco mild green mosaic virus is the active ingredient of SolviNix (BioProdex, Gainesville, FL), currently sold in United States for selective control of tropical soda apple (Charudattan and Hiebert, 2007; Charudattan *et al*., 2009). Plant viruses are also sources of biomaterials and nanotechnology tools, which are summarized in Table 2 and have been extensively reviewed elsewhere (Steele *et al*., 2017; Wen and Steinmetz, 2016). Engineered capsid proteins were used to enhance bioavailability of toxins and small molecules to control insects and nematodes (Bonning *et al*., 2014; Cao *et al*., 2015).

**Table 2:** Biotechnology applications of plant viruses

Use	Description	References
Enhanced plant aesthetics	Increase beauty and commercial value of ornamental plants	Valverde *et al*. (2012)
Cross‐protection	Delivery of mild virus strains to prevent infections by their severe relatives	Ziebell and Carr (2010)
Weed biocontrol	Viruses triggering lethal systemic necrosis as bioherbicides	Harding and Raizada (2015)
Pest biocontrol	Enhanced toxin and pesticide delivery for insect and nematode control	Bonning *et al*. (2014); Cao *et al*. (2015)
Nanoparticle scaffolds	Virion surfaces are functionalized and used to assemble nanoparticles	Schoonen *et al*. (2015); Steele *et al*. (2017); Wen and Steinmetz (2016)
Nanocarriers	Virions are used to transport cargo compounds	Aumiller *et al*. (2018)
Nanoreactors	Enzymes are encapsulated into virions to engineer cascade reactions	Brasch *et al*. (2017); Comellas‐Aragonès *et al*. (2007)
Bioimaging	Virions are functionalized with dyes or contrast agents to enhance cell imaging	Shukla *et al*. (2013)
Recombinant protein/peptide expression	Fast, transient overproduction of recombinant peptide, polypeptide libraries and protein complexes	Dugdale *et al*. (2013); Gleba *et al*. (2014); Julve Parreño *et al*. (2018)
Functional genomic studies	Targeted gene silencing using VIGS and miRNA viral vectors	Dommes *et al*. (2018); Tang *et al*. (2010)
Genome editing	Targeted genome editing *via* transient delivery of sequence‐specific nucleases	Zaidi and Mansoor (2017)
Metabolic pathway engineering	Biosynthetic pathway rewiring to improve production of native and foreign metabolites	Bedoya *et al*. (2012); Kumagai *et al*. (1995); Majer *et al*. (2017); Mozes‐Koch *et al*. (2012); Zhang *et al*. (2013)
Flowering induction	Viral expression of *FLOWERING LOCUS T* to accelerate flowering induction and crop breeding	McGarry *et al*. (2017)
Crop gene therapy	Open‐field use of viral vectors for transient reprogramming of crop traits within a single growing season	Gleba *et al*. (2014)
Biomolecule evolution	Libraries of target sequences are cloned into viral vectors; directed *in vivo* evolution selects improved or new functions	n.r.

n.r., not reported.

Full‐length infectious clones can be engineered and optimized as viral vectors (Table 2; Figure 2). Almost four decades ago, pioneering studies demonstrated the potential of viruses as delivery vectors for transferring exogenous sequences to plants (Brisson *et al*., 1984; French *et al*., 1986; Gronenborn *et al*., 1981; Takamatsu *et al*., 1987). Since then, efforts of the scientific community established viral vectors as an alternative to stably transformed transgenic or transplastomic plants for industrial production of a wide range of pharmaceuticals (Gleba *et al*., 2014; Hefferon, 2017). By maintaining development time and costs to a fraction of those required for stable transformation, the combination of *Agrobacterium* and viral vectors maximizes flexibility, scalability and yields. *Agrobacterium*‐based expression has proven the most significant progress in the manufacturing of plant‐made proteins and compounds (Marillonnet *et al*., 2005; Peyret and Lomonossoff, 2015). Apart from their use for protein expression, viral vectors are also widely applied in functional genomic studies for targeted down‐regulation of endogenous transcripts *via* RNA silencing (Dommes *et al*., 2018; Tang *et al*., 2010). Stable genome alterations in plant cells have been reported by viral expression of sequence‐specific nucleases or use of virus‐delivered sequences as template for targeted DNA replacement (Ali *et al*., 2015; Baltes *et al*., 2014; Gil‐Humanes *et al*., 2017; Honig *et al*., 2015; Marton *et al*., 2010; Wang *et al*., 2017). Once editing efficiency and heritable transmission of modified alleles are optimized, virus‐mediated genome editing would bypass requirements for plant transformation and regeneration, thus expediting the engineering and breeding of new crop varieties. Lack of exogenous sequences would make genome‐edited plants indistinguishable from those obtained by traditional mutagenesis methods and potentially avoid the need for regulatory approval (Globus and Qimron, 2018). Regulatory procedures nonetheless vary from country to country, and a setback has arisen from a recent European Union court ruling stating that gene‐editing technologies are subject to all obligations of genetically modified organisms (Callaway, 2018).

An exciting and promising approach to tackling time‐consuming breeding schemes and regulatory approval procedures would be the use of virus‐based vectors as gene therapy tools for existing crop varieties. Recombinant viral vectors are common means to genetically reprogramme mammalian cells for basic research and therapeutic purposes. Engineered human viruses have proven to be effective oncolytic agents and delivery vehicles for gene therapy and genetic circuitries, and are studied in an increasing number of clinical trials (Kaufman *et al*., 2015; Kotterman *et al*., 2015; Nissim *et al*., 2017). In 2005, a genetically modified adenovirus was approved as an oncolytic drug for cancer treatment. In 2012, an adeno‐associated virus‐based vector was approved for gene therapy treatment. On these same lines and as suggested by Gleba *et al*. (2014), what if we could use engineered plant viruses as gene therapy tools to reprogramme field‐grown crops? As proof of principle, viral vectors have been used to rewire plant biosynthetic pathways by delivery of transcription factors, targeted knock‐down of metabolic genes or overexpression of heterologous enzymes (Bedoya *et al*., 2012; Kumagai *et al*., 1995; Majer *et al*., 2017; Mozes‐Koch *et al*., 2012; Zhang *et al*., 2013). Such approaches might be applied for crop biofortification as an alternative or in conjunction to standard breeding and transgenic strategies. Secondary metabolites can also act as volatile signals (semiochemicals) for other members in the phytobiome (Pickett and Khan, 2016). Plant viruses might be exploited for pest control of crops by engineering production of semiochemicals that act as pest repellents or recruiting signals for predators and parasitoids. Complex physiological and developmental traits can be reprogrammed using engineered viruses and comprehensive knowledge of the molecular biology and genetics of model plants. Virus‐induced gene silencing can be used to suppress negative regulators of desired crop traits and, in a complementary approach, positive regulators can be ectopically expressed using viral vectors. One of the most successful examples of the latter is control of flowering through viral overexpression of the *FLOWERING LOCUS T* (*FT*) gene, an approach termed virus‐induced flowering that was first reported in cucurbits (Lin *et al*., 2007). Flowering induction in tree species with a long juvenile phase may take years or decades. Virus‐induced flowering promotes FT accumulation, early flowering and has been applied to accelerate genetic studies and breeding programmes of cotton, citrus and apple (McGarry *et al*., 2017; Velázquez *et al*., 2016).

Engineering of complex regulatory circuits and traits might require the use of multiple components. Viral vectors permit co‐expression of various proteins that can be targeted to diverse subcellular compartments (Majer *et al*., 2015). Multiple proteins have been expressed simultaneously using single viral vectors. Genes of interest are placed under the control of independent subgenomic promoters or expressed as large polyproteins that are processed post‐translationally by virus‐encoded proteases or self‐cleaving 2A peptides to release functional subunits. Subgenomic segments of multipartite viruses can be modified to host expression cassettes (Figure 1b). Systems based on multipartite viruses and helper viruses/satellites have been reported for protein co‐expression and dual gene silencing (Cheuk and Houde, 2018; Liou *et al*., 2014, 2017; Peyret and Lomonossoff, 2015).

## Synthetic virus populations and consortia

**Figure 3:**
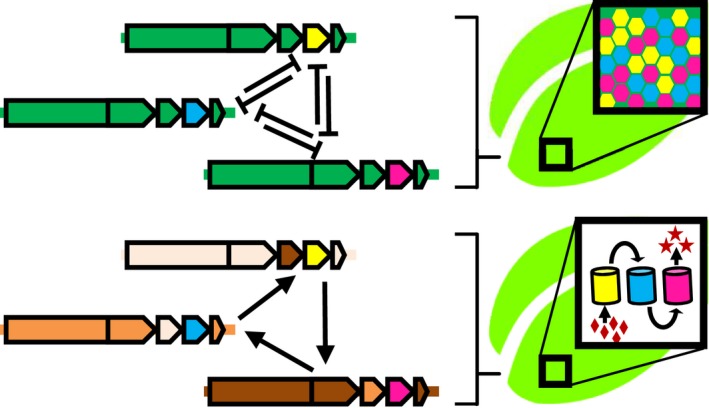
Engineering synthetic virus populations and consortia. Viral vectors for co‐expression of various heterologous proteins (in cyan, yellow and magenta). Top, functions of antagonistic viruses can be compartmentalized by superinfection exclusion events. Bottom, individual functions of cooperating viruses can synergize and give rise to consortium functions; virus interdependencies might also be engineered as biocontainment systems.

Plant viruses can exist as populations, and mixtures of different species are common in nature (Elena *et al*., 2014; Pagán and García‐Arenal, 2018). The use of synthetic multispecies communities is an emerging trend to augment microbiome systems for industrial and environmental biotechnology (Johns *et al*., 2016). Simultaneous delivery of viruses with specialized tasks can additively give rise to population functions that are simply more efficient or could be difficult or impossible to achieve otherwise (Figure 3). Features of synthetic virus communities might include ease of assembly and optimization of individual subsystems as well as the possibility to diversify and compartmentalize functions. Low cargo capacity is often seen as a major constraint of viral vectors. Large multicomponent sequences can be divided across different vectors to reduce individual genomic loads and burden. In plants, a combination of noncompeting viral vectors was shown to drive high‐yield expression of hetero‐oligomeric protein complexes that cannot be obtained with a single viral vector (Giritch *et al*., 2006). Although co‐infecting viruses can coexist and cooperate, antagonism is also a common behaviour known as superinfection exclusion. This event often occurs in co‐infections of phylogenetically related viruses and results in somatic mosaics (individual clone compartmentalization into defined plant cell clusters; Figure 3). Very recently, the superinfection exclusion phenomenon was leveraged by a virus population approach to boost recombinant polypeptide diversity and recover hundreds of variants from plants (Julve Parreño *et al*., 2018). While designing synthetic virus populations and communities with predictable outputs poses outstanding challenges, its implementation has the potential to be a disruptive advance in plant synthetic biology and crop engineering.

**Figure 4:**
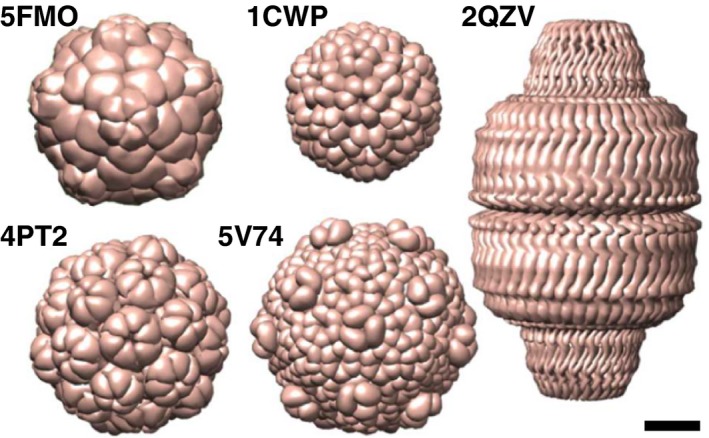
Virus‐mediated engineering of biocompartments. Examples of protein shells for *in vivo* biocompartment engineering; protein data bank (PDB) accessions are indicated: 5FMO, virus‐like particles of cowpea mosaic virus; 1CWP, cowpea chlorotic mottle virus virion; 4PT2, *Myxococcus xanthus* encapsulin protein; 5V74, *Haliangium ochraceum* microcompartment shell; 2QZV, rat vault shell. Molecule surfaces were rendered in Chimera (Pettersen *et al*., 2004); scale bar, 10 nm.

Increased genetic loads of virus consortia would make it feasible to transiently deliver entire heterologous or synthetic pathways to plants (Figure 3). In bacteria, a variety of metabolic processes are compartmentalized in microcompartments with semipermeable protein shells (Kerfeld *et al*., 2018). The confined microenvironment improves metabolic flux by intermediate trapping, enzyme crowding and protection; this is also thought to prevent unwanted side reactions and release of toxic metabolic intermediates. Capsids from plant viruses have been repurposed as *in vitro* nanoreactors (Table 2), that is, protein shells for enzyme‐catalysed cascade reactions (Brasch *et al*., 2017; Comellas‐Aragonès *et al*., 2007). To further expand the design space and given their genetic modularity, plant viruses can be used to express protein scaffolds found in other biosystems (Pieters *et al*., 2016) (Figure 4). For instance, vault and encapsulin nanoparticles assemble in heterologous eukaryotic systems when expressed using viral vectors (Rome and Kickhoefer, 2013; Sigmund *et al*., 2018). Shell proteins of cyanobacterial carboxysomes can self‐assemble into organized structures once transiently expressed in plants by agro‐infiltration (Kerfeld *et al*., 2018; Lin *et al*., 2014). Use of viral vectors and virus consortia for *in vivo* engineering of biocompartments could provide a way to enhance photosynthetic performance and other metabolic traits of crops.

Synthetic plant virus populations could also be used to obtain biomolecules with improved or new functions by virus‐directed evolution. Phage‐assisted continuous evolution (PACE) was successfully applied in bacteria to develop tailor‐made protein‐nucleic acid and protein–protein interactions (Brödel *et al*., 2018). Such a virus–bacterium system was exploited to evolve *Bacillus thuringiensis* δ‐endotoxins with enhanced insecticidal potency and agricultural utility (Badran *et al*., 2016). Protein solubility, folding, posttranslational modifications and the biochemical context could limit PACE applicability for eukaryotic proteins. The design of suitable virus–plant methods would permit to evolve plant‐specific biomolecules (e.g. photosynthetic, membrane proteins) not amenable to bacterial expression and selection.

## Biocontainment strategies for the real world

Containment of plant viruses in laboratories and closed facilities can be achieved by specific regulatory frameworks that include physical and logistical barriers as well as staff training, which have been reviewed recently (Brewer *et al*., 2018). Notwithstanding, a major goal of synthetic biology is to build engineered organisms with improved or new functions and applications that can solve real‐world needs (Liu and Stewart, 2015). Advanced biocontainment systems have been designed to control microorganism escapees (Johns *et al*., 2016; Lee *et al*., 2018).

The aim for applied research and future commercial uses of engineered plant viruses calls for innovative biodesigns that limit agricultural and environmental risks. In plants, current approaches to avoid unwanted spread of viral vectors include the mutagenesis or deletion of genes necessary for virus transmission. Potyvirus expression vectors with coat protein mutations that impede aphid transmissibility have been reported, and tobravirus‐based vectors often lack nematode transmission genes (Peyret and Lomonossoff, 2015; Touriño *et al*., 2008). Additional non‐essential virus genes with undesired functions can be removed in a deconstructed virus strategy to increase expression efficiency, control and overall safety. Safety can be further improved by eliminating essential genes needed for virus amplification and systemic spread. Activation of defective viral vectors was reported through the controlled use of recombinases, splicing or in transgenic hosts that provide the essential gene functions in *trans* (Dugdale *et al*., 2013; Fukuzawa *et al*., 2011; Gleba *et al*., 2004; Marillonnet *et al*., 2004). To overcome needs of transgenic hosts, this concept can be superseded by design of synthetic virus communities in which viral clones are forced to coexist by engineered interdependencies (Figure 3, bottom). For instance, systemic spread of defective clones can be restored by community members that express movement proteins with orthogonal functions. Reduced escapee rates are predicted, since unwanted virus releases would be possible only by simultaneous escapes of all the community members or by breaking community rules (e.g. restoring recombination).

Virus containment can be regulated conditionally by supplying chemical compounds or unnatural genetic elements (Kelemen *et al*., 2018). Insertion of non‐sense and unnatural codons into essential virus genes can condition the replication of engineered viruses to the presence of specific chemical and genetic suppressors. Unnatural amino acids and quadruplet codons were used to generate live replication‐incompetent viruses as safer vaccines (Chen *et al*., 2018; Si *et al*., 2016). In plants, viral expression of the cytochrome P450_SU1_ conferred conditional plant sensitivity to the R7402 proherbicide (Whitham *et al*., 1999). Similar approaches can be used to negatively select infected plants and control escapees. A removable RNA virus vector was generated by including a target site of an inducible endogenous miRNA (Chujo *et al*., 2017). Organ‐specific miRNAs can be used to selectively deplete viral vectors. Topically applied double‐stranded RNA molecules represent an emerging, attractive alternative for control of plant viruses that could be adapted for the targeted removal of specific viral vectors (Mitter *et al*., 2017). Public acceptance of crop gene therapies might be fostered by biodesigns that improve overall plant fitness while yielding virus‐free fruits, seeds and other edible parts.

Use of *Agrobacterium* for viral vector delivery would require release of engineered bacteria into the environment. Fortunately, *Agrobacterium* is ubiquitous in field soils, and auxotrophic strains have been generated that need exogenous metabolite supplies and are unable to survive in nature (Collens *et al*., 2004; Marillonnet *et al*., 2012; Ranch *et al*., 2012). Programmable biocontainment circuits that control bacterial survival by conditionally activating toxin expression or repressing essential genes reduce escapee rates by several orders of magnitude (Lee *et al*., 2018). Implementation of kill switches in *Agrobacterium* cells could provide an additional layer of biocontainment.

## Future perspectives

To make the most of metagenomic data, solid workflows are needed to set up reverse genetic systems for plant viruses. Conversely, metagenomics and high‐throughput sequencing technologies facilitate building of reliable study systems for known viruses. *Agrobacterium*‐mediated infection provides a simple, convenient and efficient method already used for many plant viral and subviral agents. Its potential and throughput can be greatly increased by adoption of synthetic biology strategies for assembly of binary infectious clones. Obtaining infectious clones of double‐ and negative‐stranded RNA viruses is still a challenge (Figure 1c). Reports of success for human viruses (Desselberger, 2017; Mogler and Kamrud, 2015) and, more recently, for plant viruses (Ishibashi *et al*., 2017; Wang *et al*., 2015) indicate that establishing reverse genetics systems for major plant viruses is just a matter of time. Current DNA synthesis advances allow recreation in principle of all known (plant) viruses and pave the way towards manufacturing computationally designed synthetic systems, including artificial viruses and microbiomes (Butterfield *et al*., 2017; Johns *et al*., 2016).

Knowledge of virus genetics and biology contributed to the foundations of modern plant molecular biology and biotechnology. In the same way, the systemic analysis of virus–phytobiome interactions would provide valuable resources for plant fundamental and applied research. In plants, approaches based on viral vectors or virus‐derived components have been used for a wide range of applications − pharmaceutical production, high‐density fruit tree plantings, weed and pest biocontrol, metabolic pathway and circuit engineering, plant genomic studies, targeted editing and *ad hoc* flowering induction, to name a few. The coupling of *Agrobacterium*‐mediated delivery and virus vectors prompted a leap in flexibility and scalability of plant expression systems. To better understand how the power of agro‐infection could affect the future of plant biotechnology, readers are invited to refer to an illuminating review by Gleba and coworkers (Gleba *et al*., 2014). In optimal agro‐infection conditions, as few as eight *Agrobacterium* cells (and possibly a single cell of the supervirulent CryX strain) are needed to initiate viral replication (Marillonnet *et al*., 2005; Roemer *et al*., 2015). Plant delivery of collections of virus species and genome variants can be achieved easily and efficiently by bacteria pooling. These notions open a new dimension for virus genomic and virus–virus interaction studies and have already spurred the development of the first biotechnological applications of synthetic virus populations.

Considering the agro‐infection potential, successes and acceptance of virus‐based therapies for clinical uses, it seems reasonable to imagine a future in which plant viruses are applied in crop gene therapies. A growing number of agrochemical and rapidly emerging venture‐backed companies are centring their attention on repurposing plant‐associated microbes to replace chemical fertilizers and pesticides, and improve crop stress tolerance and yield (Brophy *et al*., 2018; Mueller and Sachs, 2015; Waltz, 2017). Advantages of engineered viruses include easier, faster component characterization, which can reduce costs of technology development and at the same time improve flexibility, to create new products and crop traits as well as to meet evolving market needs. Complex traits could be engineered using virus populations and consortia as platforms for the delivery of sequence‐specific editors and transcriptional modulators, genetic circuits or biosynthetic pathways (Kassaw *et al*., 2018; Knott and Doudna, 2018; Mahas *et al*., 2018; Murovec *et al*., 2017; Wurtzel and Kutchan, 2016). Regulatory requirements and obstacles for commercial use of crop gene therapies that employ viral vectors are still unclear. The legislative burden for approval of engineered microorganisms might be significantly lower than that for genetically modified plants. Of note and likely applicable to viral vectors in general, possible stances of regulatory agencies on the agronomical use of the virus‐induced flowering system were recently analysed (McGarry *et al*., 2017).

**Figure 5:**
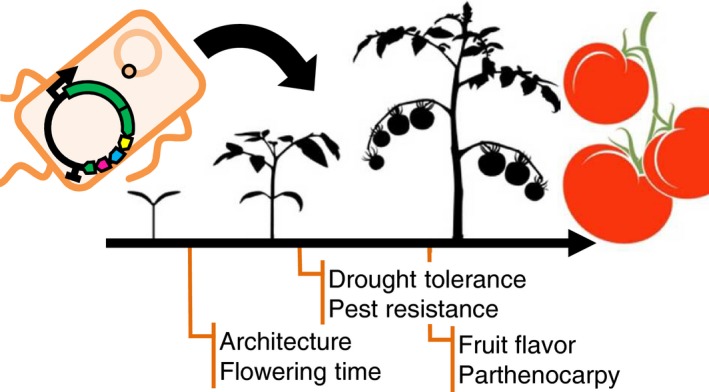
Virus‐based gene therapies for crops. Agro‐infection of engineered viruses might be used for *ad hoc*, transient reprogramming of field‐grown plants. The tomato production cycle and representative traits that might be altered by use of viral vectors are shown.

Innovative biocontainment solutions that remove or severely narrow chances of escapes and natural ecosystem risks would probably maximize benefits to society and its acceptance of engineered viruses for agricultural and horticultural uses. In the short term, whereas approval for open‐field use of viral vectors appears unrealistic until robust biocontainment solutions are devised, we can envisage specialized greenhouse facilities applying gene therapies to high‐value horticultures (Figure 5). For instance, achievements in plant genetics can immediately be leveraged to design virus‐based therapies to enhance tomato productions (Azzi *et al*., 2015). In tomato, customized control of plant architecture, fruiting precocity, fruit flavour, parthenocarpy, drought tolerance and whitefly resistance can be achieved by virus‐mediated regulation of branching factors (Martín‐Trillo *et al*., 2011), flowering induction (Lifschitz *et al*., 2006), organoleptic compound contents (Tieman *et al*., 2017; Zhu *et al*., 2018), sporocyte development (Rojas‐Gracia *et al*., 2017), abscisic acid signalling (González‐Guzmán *et al*., 2014) and trichome density (Firdaus *et al*., 2012) respectively.

Virus genomes provide an excellent background in which to mine previously unknown molecular tools and elements for genetic circuitries. Viruses and their components have been domesticated by prokaryotic and eukaryotic hosts for new cellular roles unrelated to the original functions, and recruited in processes as disparate as host defence, insect parasitization, animal placentation and neuronal communication (Koonin and Krupovic, 2018). Novel virus‐based applications will likely be conceived with the uncovering of new cases of virus exaptation. Finally, it is predicted that increasing scalability of virus reverse genetic systems will improve our ability to document plant–virus and virus–virus interaction outcomes, to learn valuable lessons on virus and plant biology and to design biotechnological applications for generation of plants with reliable and precise functions, better physiological and agronomic traits or new product engineering.

## Author contributions

F.P. conceived and drafted the manuscript; W.M. prepared Box 1 contents; J.‐A.D. collaborated in the manuscript preparation. All authors revised and approved the final manuscript.
